# Simplifying Preclinical Periodontal Instrumentation Assessment: A Pass/Fail Rubric Approach

**DOI:** 10.1002/jdd.70052

**Published:** 2025-09-30

**Authors:** Sheryl Syme, Oksana Mishler, Christine Barnes, Se‐Lim Oh

**Affiliations:** ^1^ Department of Advanced Oral Sciences and Therapeutics University of Maryland School of Dentistry Baltimore Maryland USA

## 1 Problem

Traditional score‐based evaluations in preclinical periodontal instrumentation at the University of Maryland School of Dentistry have shown consistent failure rates of 19% and considerable faculty time spent on remediation. While score‐based assessments aimed to rigorously measure technique fidelity, they may inadvertently induce negative educational outcomes, including elevated student anxiety, instructional inefficiency, and uncertain predictivity for future clinical performance. In response, faculty engaged in evidence‐based revisions to better align teaching and assessment practices with the clinical competency outcomes essential for quality patient care [1]. Grounded in Messick's framework of consequential construct validity, which asserts that assessment legitimacy hinges not only on psychometric rigor but also on its educational impact [2], this report evaluated whether a simplified pass/fail rubric can uphold clinical competency standards while enhancing instructional effectiveness.

## 2 Solution

**FIGURE 1:**
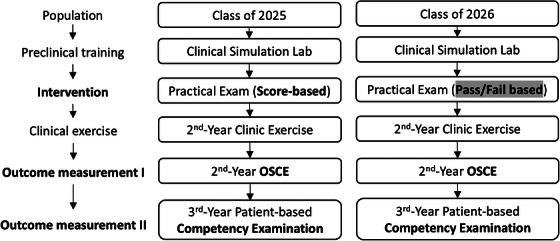
A chart of study flow.

To address persistent challenges with a traditional score‐based evaluation, faculty redesigned the assessment for the Class of 2026 in their second‐year preclinical periodontal instrumentation practical examination. A simplified pass/fail rubric (Table 1) was developed by eliminating all points assigned and all technical aspects from the previous score‐based rubric through consensus, focusing on critical performance criteria—identifying the correct instrument with the correct working end, which is consistent with literature discussing impact of pass/fail grading systems [3, 4, 5]. This binary (pass or fail) rubric aimed to streamline evaluations, reduce faculty workload, and foster a more supportive learning environment. No changes were made to the instructional content or flow (Figure 1). Data were collected over two academic years and analyzed using Chi‐square statistics (Table 2), comparing outcomes on the second‐year practical, second‐year objective structured clinical examination (OSCE), and the third‐year patient‐based instrumentation competency examination between the Class of 2025 (score‐based rubric) and Class of 2026 (pass/fail rubric).

**TABLE 1:** Simplified Pass/Fail‐based rubric from score‐based Practical Examination Rubric.

**Gracey 11/12** (20 points)	
**Critical Failure** Incorrect instrument used Incorrect working end used Using the instrument on the distal surface of the tooth Scaling “backwards” Not performed on the palatal side	□ Inappropriate pen grasp (−2) □ No/inappropriate fulcrum (−2) □ Incorrect insertion point (−2)
	□ Overall stroke was too weak/ little pull (−2) □ Little/no overlapping stroke/not continuous (−2) □ Not going interproximally (−2) □ Not enough rolling of the tip of the instrument (−2) □ Instrument adaptation is too open/closed (−2)
**Gracey 13/14** (20 points)	
**Critical Failure** Incorrect instrument used Incorrect working end used Using the instrument on the mesial surface of the tooth Scaling “backwards” Not performed on the palatal side	□ Inappropriate pen grasp (−2) □ No/inappropriate fulcrum (−2) □ Incorrect insertion point (−2)
	□ Overall stroke was too weak/ little pull (−2) □ Little/no overlapping stroke/not continuous (−2) □ Not going interproximally (−2) □ Not enough rolling of the tip of the instrument (−2) □ Instrument adaptation is too open/closed (−2)
**Universal Columbia 13/14** (20 points)	
**Critical Failure** Incorrect instrument used Incorrect working end used Flipping the instrument on the same tooth surface Scaling only the half surface on the tooth Scaling “backwards” Not performed on the palatal side	□ Inappropriate pen grasp (−2) □ No/inappropriate fulcrum (−2) □ Incorrect insertion point (−2)
	□ Overall stroke was too weak/ little pull (−2) □ Little/no overlapping stroke/not continuous (−2) □ Not going interproximally (−2) □ Not enough rolling of the tip of the instrument (−2) □ Instrument adaptation is too open/closed (−2)

*Note*: The red font indicates the deletions from the previous score‐based rubric. The new rubric included only the black font.

**TABLE 2:** Summary of student performances in each examination.

		Class of 2025 (*n* = 127)	Class of 2026 (*n* = 138)	Total (*N* = 265)	*p* value^1^
Second Year practical examination	Assessment	Score‐based	Pass/fail based		0.025
	Pass	112 (88.2%)	132 (95.7%)	244 (92.1%)	
	Fail	15 (11.8%)	6 (4.3%)	21 (7.9%)	
Second Year OSCE	Universal				0.702
	Pass	115 (90.6%)	123 (89.1%)	238 (89.9%)	
	Fail	12 (9.4%)	15 (10.9%)	27 (10.2%)	
	Gracey 13/14				0.229
	Pass	109 (85.8%)	125 (90.6%)	234 (88.3%)	
	Fail	18 (14.2%)	13 (9.4%)	31 (11.7%)	
	Gracey 11/12				0.905
	Pass	123 (96.9%)	134 (97.1%)	257 (97%)	
	Fail	4 (3.1%)	4 (2.9%)	8 (3%)	
Third Year	Instrumentation Competency Examination				0.087
	Pass	107 (84.3%)	128 (92.8%)	235 (88.7%)	
	Fail once	17 (13.4%)	9 (6.5%)	26 (9.8%)	
	Fail twice	3 (2.4%)	1 (0.7%)	4 (1.5%)	

1 Chi‐squared test.

## 3 Results

### 3.1 What Went Well

Formal faculty calibration sessions for the pass/fail‐based rubric were not conducted as there was little gray areas in the rubric; instead, brief reviews occurred before the practical examination. The Class of 2026 showed a significantly lower failure rate on the practical examination compared to the Class of 2025 (6 vs. 15 failures; *p* = 0.025). Feedback was primarily given for the failed students. Faculty remediation time decreased from 6 to 2 hours. No significant differences were observed in the subsequent second‐year OSCEs or third‐year clinical instrumentation examinations (*p* > 0.05) between the two classes, indicating that the revised rubric maintained clinical competency while reducing load for faculty and students.

### 3.2 Lessons Learned

These findings suggest that simplifying the rubric did not compromise clinical readiness. The simplified pass/fail approach may help reduce performance‐related student anxiety by avoiding the dissection of every procedural step, as existing literature reports that overly detailed assessments can elevate stress [3, 4]. The assessment using the simplified rubric reduced failure rates and remediation time, aligning with Messick's consequential validity [2] by minimizing adverse outcomes without sacrificing standards. The binary format offered clear decision points, reduced ambiguity since no gradations or subjective partial credits were present, and therefore, supported competency‐based education. While long‐term predictive validity is in progress, these early results indicate that the simplified rubric maintains essential competencies. Robust studies with larger cohorts and longitudinal designs are needed to comprehensively evaluate the long‐term educational effectiveness of this intervention.

## Ethics Statement

This report was conducted under an approved protocol by the Institutional Review Board at the University of Maryland, Baltimore (HP‐00104016).

## Conflicts of Interest

The authors declare no conflicts of interest.
